# Detection of AZFc gene deletion in a cohort of Egyptian patients with idiopathic male infertility

**DOI:** 10.1186/s43141-023-00584-9

**Published:** 2023-11-10

**Authors:** Maha M. Eid, Ola M. Eid, Amany H. Abdelrahman, Islam F. S. Abdelrahman, Elshaimaa A. F. Aboelkomsan, Rania M. A. AbdelKader, Mirhane Hassan, Marwa Farid, Alshaymaa A. Ibrahim, Safa N. Abd El-Fattah, Rana Mahrous

**Affiliations:** 1 Human Cytogenetic Department, Human Genetics and Genome Research Institute, National Research Center, Bohouth Street, 12311 Dokki, Cairo, Egypt; 2 Clinical and Chemical Pathology Department, Medical Research and Clinical Studies Institute, National Research Center, Cairo, Egypt; 3 Andrology Department, Faculty of Medicine, Cairo University, Giza, Egypt; 4 Pathology Department, School of Medicine, New Giza University, 6th of October City, Egypt

**Keywords:** AZFc deletion, Idiopathic male infertility, Multiplex ligation-dependent probe amplification (MLPA), Azoospermia, Oligozoospermia

## Abstract

**Background:**

The deletions of azoospermic factor regions (AZF) are considered risk factor of spermatogenic failure. AZF duplications or complex copy number variants (CNVs) were rarely studied because STS-PCR could not always detect these changes. The application of multiplex ligation-dependent probe amplification (MLPA) as a valuable test for detection of the deletion and or duplication was introduced to investigate the AZF sub-region CNVs. The MLPA technique is still not applied on a large scale, and the publications in this area of research are limited. The aim of this work was to evaluate the efficacy of MLPA assay to detect AZF-linked CNVs in idiopathic spermatogenic failure patients and to evaluate its importance as a prognostic marker in the reproduction outcome.

**Results:**

Forty infertile men (37 with azoospermia and 3 with severe oligozoospermia) and 20 normal fertile men were subjected to thorough clinical, pathological, and laboratory assessment, chromosomal study, MLPA, STS-PCR assays, histopathology study, and testicular sperm retrieval (TESE). Out of the 40 patients, 7 patients have shown CNV in the AZFc region, 6 patients have partial deletion, and one patient has partial duplication. Only one of the normal control has AZFc duplication. STS-PCR was able to detect the deletion in only 4 out of the 7 positive patients and none of the control.

**Conclusion:**

We concluded that MLPA should be applied on a larger scale for the detection of Y chromosome microdeletion as a rapid, efficient, and cheap test.

## Background

Male infertility alone was found to be responsible for the failure of pregnancy in 20 to 30% of couples [1]. The deletions of the azoospermia factor (AZF) on the long arm of the Y chromosome are considered second cause for male infertility following Klinefelter syndrome [2]. This area harbors three main spermatogenesis loci, namely AZFa, AZFb, and AZFc [3].

Deletion and/or duplication involving AZF sub-regions are reported to be associated with an increased risk of reproduction and spermatogenic failure (SF) [3, 4]. Deletions within the azoospermic factor region on Y chromosome are present in 5% and 10% of severe oligospermic and azoospermic men, respectively. These microdeletions are classified according to the position of the deleted segment which is identified as AZFa (the most proximal segment), AZFb (middle), and AZFc (distal). The reported prevalence of AZF deletions within the world’s populations of infertile men is very heterogenous, ranging from < 2 to > 24% based on the origin of the studied group. AZFc deletion is the most commonly identified, and it provides for better chance for reproduction outcome through artificial reproductive techniques (ART). Conversely, deletions detected in the sub-regions of AZFa, AZFb, or any combination of regions containing these segments, are associated with poor reproduction outcome [5, 6]. This may be attributed to the non allelic homologous recombination events that occurred in these sub-regions due to the presence of several locus-specific repeats [5, 7]. Deletions affecting these regions are known to cause SF by changing the copy number of genes such as DAZ, CDY2, and BPY2 involving in spermatogenesis process [8].

Deletions at AZFc sub-region are reported to be the most common type that subsequently lead to deficiency of DAZ gene [7, 9]. Deletion of gr/gr region was considered as a risk factor for SF caused by the absence of the proximal part of the AZFc region, which is important for the normal spermatogenesis [10]. However, partial deletion of AZFc occurred in most cases that leads to reduction of the number of the DAZ genes by removing two of them [10, 11].

The rate and pathogenic effects of these deletions were found to be variable among different ethnic groups [10, 12]. Other types of CNVs in AZF regions, such microduplications and complex deletion–duplication rearrangements, were also noticed to be associated with SF [13, 14].

The conventional technique that was used to identify AZF deletion was depended on polymerase chain reaction (PCR) of sequence-tagged site (STS) markers (STS-PCR); however, this approach could not determine other CNVs such as duplication or deletion duplication which is considered as a limitation of this approach. Although they are not as common as deletion, AZF-related duplications or complex CNVs have been reported [14–16].

With the development of other molecular procedures, such as multiplex ligation-dependent probe amplification (MLPA) and array comparative genomic hybridization (aCGH), multiple CNVs have been identified using single procedure. However, the MLPA procedure was relatively simple and not expensive, and it was recommended as an informative tool for the detection of microdeletions or microduplication in many genetic diseases [17–19].

The objectives of this study are to investigate the copy number variation of AZF sub-regions in idiopathic male infertility using MLPA procedure and to evaluate the efficacy of this approach as a useful and valuable test for the detection of copy number variation in AZF regions.

## Patients and methods

### Patients

The study included 40 patients with idiopathic non obstructive azoospermia. Thorough clinical examination history taking and semen analysis were done. Patients with past medical history of undescended testes, patients who had mumps orchitis or previous exposure to chemo-radiotherapy or cytotoxic drugs, patients with clinical varicocele, patients with abnormal karyotyping as Klinefelter syndrome or XX male, and patients with normal spermatogenesis in testicular histopathology were excluded from the study.

The participating patients were scheduled for micro surgical testicular sperm extraction (TESE). Micro surgical testicular sperm extraction was performed under local/sedation anesthesia. The testicular histopathological classification was done according to Cooperberg et al. [20] into normal spermatogenesis, hypospermatogenesis, spermatogenic arrest either complete or incomplete, Sertoli cell only either complete or incomplete, and tubular hyalinization either complete or incomplete.

Also, the study included 20 normal male fathering at least one child as control group.

### Methods

#### Sample collection and DNA extraction

At least 5 mL blood were withdrawn under the complete aseptic condition from each subject on PAX gene tubes for DNA extraction. DNA extraction from the blood was done using PAXgene Blood DNA Kit according to the manufacturer’s instruction. Then the quality and quantity of the DNA samples were determined using the NanoDrop spectrophotometer.

#### MLPA using probe MLPA mix P360 Y microdeletion (MRC Holland)

MLPA was done according to the manufacturer’s instruction of (MRC-HollandProbemix P360 Y microdeletion); DNA denaturation and overnight MLPA probemix hybridization steps were followed by probe ligation and amplification on the following day. The amplified products were separated using an ABI 3500 Genetic Analyzer (Applied Biosystems, USA). The results were interpreted using Cofalyser.Net software (MRC- Holland).

#### STS-PCR assay

DNA amplification by multiplex PCR was performed using STS primers for the AZFa sub-region (sY84 and sY86), the AZFb sub-region (sY127 and sY134), the AZFc sub-region (sY254 and sY255), and the SRY gene (sY14 AND sY81). The selected primers were designed for detecting Y chromosome microdeletion according to Atia et al. [21] (Table 1).

**Table 1:** The STS primer sets used in detecting Y chromosome microdeletions

AZFa	sY84	F: 5′- AGAAGGGTCTGAAAGCAGGT -3′
		R: 5′- GCCTACTACCTGGAGGCTTC -3′
	sY86	F: 5′- GTGACACACAGACTATGCTTC -3′
		R: 5′- ACACACAGAGGGACAACCCT -3′
AZFb	sY127	F: 5′- GGCTCACAAACGAAAAGAAA -3′
		R: 5′- CTGCAGGCAGTAATAAGGGA -3′
	sY134	F: 5′- GTCTGCCTCACCATAAAACG -3′
		R: 5′- ACCACTGCCAAAACTTTCAA -3′
AZFc	sY254	F: 5′- GGGTGTTACCAGAAGGCAAA -3′
		R: 5′- GAACCGTATCTACCAAAGCAGC -3′
	sY255	F: 5′- GTTACAGCATTCGGCGTGAT -3′
		R: 5′- CTCGTCATGTGCAGCCAC -3′
SRY gene	sY14	F: 5′- GAATATTCCCGCTCTCCGGA -3′
		R: 5′- GCTGGTGCTCCATTCTTGAG -3′
	sY81	F: 5′- AGGCACTGGTCAGAATGAAG -3′
		R: 5′- AATGGAAAATACAGCTCCCC-3′

Multiplex PCR reactions were carried out in a total volume of 50 μL. Amplifications were carried out on a thermocycler (Eppendorf, Germany) with cycling conditions as follows: initial denaturation at 94 °C for 5 min, followed by 32 cycles of 94 °C for 30 s, 57 °C for 30 s, and 72 °C for 90 s, with a final extension at 72 °C for 7 min.

## Results

The study included 40 males with idiopathic infertility and 20 normal males fathering at least one child as a control group. The age of the patients ranged from 29 to 56. The duration of infertility ranged from 1 to 27 years. Thirty-seven patients (92.5%) suffered from azoospermia, and 3 patients (7.5%) have severe oligospermia.

According to the histopathological study, 22 patients have Sertoli cell only syndrome (SCO) (55%), 3 patients (7.5%) have incomplete (SCO), 12 patients (30%) have spermatogenic arrest, and 3 patients (7.5%) have incomplete hyalinization.

The testicular sperm extraction (TESE) was successful in 21 (52.5%) patients, while 19 patients have negative TESE. Among the 21 patients with successful TESE, 3 patients have AZFc deletion (50%) of patients with deletion.

**Fig. 1:**
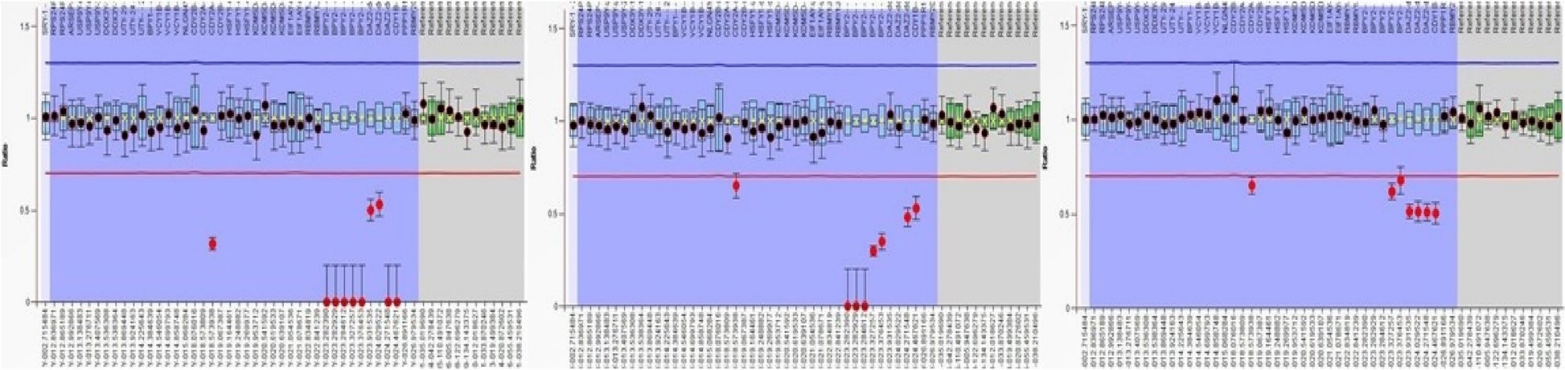
Ratio charts of MLPA results using SALSA® MLPA® Probemix P360 Y Chromosome. The charts are showing partial AZFc deletion

**Fig. 2:**
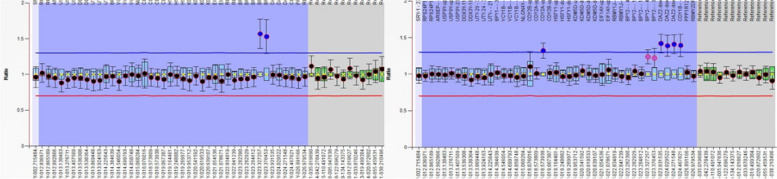
Ratio charts of MLPA results using SALSA® MLPA® Probemix P360 Y Chromosome. The charts are showing partial AZFc duplication

The MLPA results were as follows (Table 2; Figs. 1, 2): none of the patients showed deletions or duplication at AZFa or AZFb, 15% of the patients had a partial deletion at the AZFc (6 patients), only one patient has duplication at AZFc (2.5%), and the control group showed no deletion, and only one normal male had partial duplication. The summary of the genes that are frequently deleted is shown in Table 3.

**Table 2:** MLPA, PCR, and clinical data of patients with CNVs

	Age	Histopathology	TESE	Semen	MLPA	PCR
1	35	SCO	Positive	Azoospermia	Partial del	+del
2	28	SCO	negative	Azoospermia	Partial dup	-
3	34	SCO	negative	Azoospermia	Partial del	+del
4	30	SCO	negative	Azoospermia	Partial del	-
5	40	Spermatogenic arrest	negative	Azoospermia	Partial del	+del
6	36	SCO	Positive	Azoospermia	Partial del	+del
7	33	Spermatogenic arrest	Positive	Azoospermia	Partial del	-

**Table 3:** Genes frequently deleted as detected by MLPA

Gene deleted	Patient 1	Patient 2	Patient 3	Patient 4	Patient 5	Patient 6
**DAZ 2**	+	+	+	+	+	-
**BPY 2**	+	+	+	+	+	-
**CDY2A**	+	+	+	-	-	-
**(PPP1R12BP), (RBMY2DP**	-	-	-	-	-	+
**Pathology**	SCO	SCO	Spermatogenic arrest	Spermatogenic arrest	SCO	SCO
**TESE**	Positive	Negative	Negative	Positive	Positive	Negative

The STS-PCR was able to detect the deletion in only four patients (10%) and could not detect any duplications (Table 2).

## Discussion

Microdeletion of chromosome Y was considered a second common cause for male infertility following the Klinefelter syndrome [22]. The deletions of the AZF located on the Y chromosome were found to be associated with spermatogenic failure (SF). The goal of our study was to evaluate the efficacy of MLPA as a screening test for AZF CNV and if the deletion has an impact on the reproduction outcome.

We performed MLPA for 40 males with idiopathic azoospermia and oligospermia and 20 normal males. None of the patients showed deletions or duplication at AZFa or AZFb, and 15% of the patients had partial deletion at the AZFc (6 patients) and only one patient had partial duplication (1 patient) (2.5%). The control group showed no deletion, and only one male had partial duplication.

Many studies have been conducted on AZF deletions; still, there are paradoxical results of the testicular histological morphology and its correlation with the deleted AZF loci [3, 22–28]. Some reported a poor and variable correlation between the testicular histopathology and the extent of the AZF loci deletion [23], while others suggested that deletion of a particular AZF sub-region is correlated with specific testicular histopathological morphologies [3, 24]. Several authors reported that the testicular histopathological changes in patients with complete AZFa and/or AZFb microdeletion was found to affect the Sertoli cells only [25, 26]. In contrary, patients with AZFc microdeletions are presented with a wide variation of testicular morphological changes ranging from histopathological changes of the Sertoli cells only, spermatogenetic arrest, or hypospermatogenesis with the presence of sperms in semen. These findings agree with our results considering that our patients with partial AZFc deletions have either SCO or spermatogenic arrest. Also, it was suggested that the pathological changes associated with AZFc microdeletion are less severe than those associated with AZFa or AZFb [27].

Comparing our results with the previously reported data, it was found that some authors reported AZFc deletion in relative lower rate. Bunyan et al. [28] have found deletion by MLPA in 8% of the patients, but the duplication was almost the same as ours (2%). On the other hand, Franchim et al. [29] have detected deletion by MLPA in 21% of their patients, but duplication was detected in almost 7% of the patients.

The 15% for partial AZFc deletions detected in our patients is considered higher than values reported by other larger patients’ sample studies, which reported deletion rate of about 5% [15, 30]. In our study, partial duplication was seen in both test groups (1/40 infertile males and 1/20 fertile controls) which agrees with the conclusion reached by Giachini et al. [15] stating that the small duplications may not have an impact on spermatogenesis events. On the other hand, the impact of AZFc duplication is still controversial; other authors suggested that duplications may affect male fertility [31]. Furthermore, Lu et al. [8] studied the extent of the spermatogenic involvement and the presence of multiple copies ofAZFc genes by gene dosage in eight families, and they found out that only the CNVs of the DAZ and BPY2 genes were associated with spermatogenic failure. This finding may explain the infertility of our patient who had duplication involving BPY2 gene probes, but on the other hand, the normal control has duplication involving CDY2A, BPY2, and DAZ2. So, it may be due to the small number of patients reported in the literature with duplication that led to the interpretation of its impact in spermatogenesis failure to be still controversial.

It was long believed that patient with AZFb or AZFbc deletion has negative TESE [32, 33], but other authors claimed the opposite [34, 35]. Since none of our patients showed deletion or duplication involving AZFa or AZFb, we cannot evaluate this point.

The *DAZ2*, *CDY2*, and *PBY2* are the common deleted genes among our patients, and they are the same commonly reported genes deletion in the literature [35]. It was reported that these three genes are the important candidate genes in this area, and they are the key players in the process of the spermatogenesis [36, 37].

In our study, MLPA was able to detect deletion in more patients than STS-PCR which agree with other studies [28, 29]. The MLPA technique has many advantages over STS-PCR technique as it allows the detection of almost all of the possible AZF sub-regions deletion or duplication in a single reaction. MLPA was found to be the ideal procedure to detect gene dosage as reported in the literature, where it could detect single-copy deletions or duplications [28]. On the other hand, the MLPA has few limitations where the probe signal intensity may vary according to DNA purity, and this variation could be attributed to the DNA extraction protocol, elution solution, degradation degree, and presence of contaminants [38, 39].

## Conclusion

From our results and previous reports, we can conclude that P360 MLPA probemix provide accurate, cheap, and rapid test for the detection of AZF deletions. It can be used as a screening test that investigates large number of genes in one step which could be missed by the conventional STS-PCR. The application of such method on larger scale will easily lead to the accumulation of more data that may add to a better understanding of the importance of the deleted genes controlling the process of spermatogenesis.

Also, screening patient with azospermia and oligospermia to detect the presence of Y chromosome microdeletions has proven to be very important in the clinical assessment of the infertile male, as the extent of the deletions often help with the decision-making and the recommendations for the artificial reproductive technologies (ART). Among the three regions deletions, the AZFc deletion is the most frequently diagnosed, accounting for 60–80% of all reported deletions which are also associated with the highest probability of the ART successful outcome.

## Data Availability

Data and material are available upon request.
